# *Mycoplasma haemocanis* – the canine hemoplasma and its feline counterpart in the genomic era

**DOI:** 10.1186/1297-9716-43-66

**Published:** 2012-09-28

**Authors:** Naíla C do Nascimento, Andrea P Santos, Ana MS Guimaraes, Phillip J SanMiguel, Joanne B Messick

**Affiliations:** 1 Department of Veterinary Pathobiology, Purdue University, 725 Harrison Street, West Lafayette, IN, 47907, USA; 2 Purdue Genomics Core Facility, Purdue University, 170 S. University Street, West Lafayette, IN, 47907, USA

## Abstract

*Mycoplasma haemocanis* is a hemotrophic mycoplasma (hemoplasma), blood pathogen that may cause acute disease in immunosuppressed or splenectomized dogs. The genome of the strain Illinois, isolated from blood of a naturally infected dog, has been entirely sequenced and annotated to gain a better understanding of the biology of *M. haemocanis*. Its single circular chromosome has 919 992 bp and a low G + C content (35%), representing a typical mycoplasmal genome. A gene-by-gene comparison against its feline counterpart, *M. haemofelis*, reveals a very similar composition and architecture with most of the genes having conserved synteny extending over their entire chromosomes and differing only by a small set of unique protein coding sequences. As in *M. haemofelis*, *M. haemocanis* metabolic pathways are reduced and apparently rely heavily on the nutrients afforded by its host environment. The presence of a major percentage of its genome dedicated to paralogous genes (63.7%) suggests that this bacterium might use antigenic variation as a mechanism to evade the host’s immune system as also observed in *M. haemofelis* genome. Phylogenomic comparisons based on average nucleotide identity (ANI) and tetranucleotide signature suggest that these two pathogens are different species of mycoplasmas, with *M. haemocanis* infecting dogs and *M. haemofelis* infecting cats.

## Introduction

Hemotrophic mycoplasmas (hemoplasmas) are uncultivable cell-wall less bacteria, formerly classified as *Haemobartonella* and *Eperythrozoon* species, that adhere to the surface of the erythrocytes of their vertebrate hosts. These bacteria form a new clade within the *Mycoplasma* genus (class Mollicutes) and are phylogenetically related to the pneumoniae group of the mycoplasmas [1-5].

*Mycoplasma haemocanisHaemobartonella canis* was first described in Germany in 1928 in a splenectomized dog [6]. The name *Bartonella canis* was proposed and remained until 1939 when Tyzzer and Weinman created the new genus *Haemobartonella*[7]. *M. haemocanis*, proposed species name since 2002 [5], is a pleomorphic bacterium with coccoid and ring forms that can be visualized in the host’s peripheral blood smear either singly or in chains that can resemble a “violin-bow” form [8]. It may cause overt, hemolytic anemia in immunosuppressed [5,9] or splenectomized dogs [5,10], and has a worldwide distribution with prevalence of infection varying from 0.5% to 40% [11-14].

Similarities with the feline hemoplasma *M. haemofelisHaemobartonella felis*, together with the fact that hemoplasmas are not species-specific as previously thought [15,16], led some research groups to hypothesize that these two bacteria could be the same species infecting different hosts [17,18]. Moreover, there are some reports in the old literature stating that *M. haemocanis* could infect cats; however *M. haemofelis* did not cause infection in dogs [19-21]. In 1961, Dr Lumb published the manuscript “Canine haemobartonellosis and its feline counterpart”, reporting cross-transmission experiments: it was shown that when blood from cats infected with *M. haemocanis* was injected into susceptible splenectomized dogs, organisms could be seen on their peripheral blood smears [8]. It was concluded based on this evidence that the feline might act as a reservoir for *M. haemocanis*. However, blood from dogs previously injected with *M. haemofelis* inoculated into susceptible cats failed to result in circulating organisms, leading to the conclusion that these two bacteria were different species [8,22]. Forty years later, the sequences of the 16S rRNA genes of these two bacteria were reported to have 99% identity [17] raising the same question again. In 2002, the sequences of the RNase P genes of these bacteria were reported having 94.3 to 95.5% identity [18]. While the results of the RNase P genes did not support the hypothesis that *M. haemocanis* and *M. haemofelis* were identical, this additional data was still considered insufficient to determine whether these organisms should be classified as different species, subspecies, or strains of the same species [18].

Recently, three species of hemoplasmas, including *M. haemofelis*, had their genomes completely sequenced and annotated [23-27]. The aim of this study was to sequence the whole genome of *M. haemocanis* in order to better understand its biology and to perform a complete genomic comparison with its counterpart, *M. haemofelis*.

## Materials and methods

### Bacterial strain and DNA isolation

*M. haemocanis* organisms were isolated from the blood of a naturally infected dog at peak of bacteremia [17]. Written informed consent was obtained from the client for publication of this report. Bacterial genomic DNA was extracted using Quick-gDNA MiniPrep kit according to the manufacturer’s instructions (Zymo Research, Irvine, CA, USA).

### *M. haemocanis* strain Illinois sequencing and assembly

Whole genome was sequenced from paired-end libraries (TruSeq DNA sample preparation kit, Illumina, San Diego, CA, USA) using 20% of an Illumina® v3 chemistry lane (HiScanSQ). Sequencing resulted in 15.7 million high-quality filtered read pairs with an average read length of 2 × 100 nucleotides and a > 3400 X genome equivalent coverage. Reads were assembled using ABySS-PE v1.2.7 utilizing 20% of the reads with “kmer” set to 95 bases [28]. Predicted scaffolds with significant BLAST matches to canine DNA were excluded and the remaining mycoplasma scaffolds were then organized based on the orientation predicted in the assembly and on the genome sequence of *M. haemofelis* strain Ohio2. A total of 13 gaps were identified and closed using conventional PCR followed by Sanger sequencing.

### Genome annotation and analyses

First pass annotation was achieved using the NCBI annotation pipeline. Manual annotation/curation of each gene was performed using the annotation tool Manatee, provided by the Institute for Genome Sciences (IGS) at the University of Maryland, School of Medicine. Comparative analyses with other bacterial genomes were performed based on genomic data deposited in the NCBI database (NCBI, Bethesda, MD, USA).

The assignment of paralogous gene families was performed using BLASTclust tool provided by Max-Planck Institute for Developmental Biology [29], with 70% covered length and 30% sequence identity thresholds. Subcellular localization and protein sorting signals were predicted for each unique protein coding sequence (CDS) of *M. haemocanis* and *M. haemofelis* using PSORTb v.3.0 [30,31]. Metabolic pathways were predicted based on the KEGG pathway database [32] and the study reported by Yus et al. [33]. Presence of lipoproteins was predicted by LipoP version 1.0 software [34]. In addition, the tandem repeats were identified using the Tandem repeats finder program [35]. Comparative analyses of the whole genome of *M. haemocanis* and *M. haemofelis* strain Ohio2 were performed using the same tools mentioned above and all the CDSs from both genomes were evaluated using BLASTp and/or BLASTn in order to obtain a complete detailed comparison. CDSs were assigned using BLASTp and considered unique to *M. haemocanis* or *M. haemofelis* when there were no matching sequences in the aligned sequences list with ≥ 90% coverage and ≥ 30% identity or ≥ 80% coverage and ≥ 40% identity to the query sequence. Extended similarity group method for automated protein function prediction (ESG software) [36] was applied for both sets of unique CDSs.

### Species differentiation analyses

The average nucleotide identity (ANI; MUMmer algorithm) and tetranucleotide signature correlation index between genomes were calculated using JSpecies software as previously described [37]. In addition to the genome of *M. haemocanis* strain Illinois, the following genome sequences were used in the analyses: *M. haemofelis* strain Ohio2 (CP002808.1), *M. haemofelis* strain Langford (FR773153.2), *M. suis* strain Illinois (CP002525.1), and *M. suis* strain KI3806 (FQ790233.1). If two organisms had ANIm and tetranucleotide coefficients greater than 94% and 0.99, respectively, they were considered the same species [37].

## Results

### *Mycoplasma haemocanis* strain Illinois genome features

The complete singular circular chromosome of *M. haemocanis* strain Illinois has a size of 919 992 base pairs (bp) and G + C content of 35%; these genomic features are similar to other hemoplasmas species sequenced to date [23,24,26,27] and within the range reported for other members of the genus *Mycoplasma* (Table 1). As described for all sequenced mycoplasmas (24 species to date), *M. haemocanis* also uses the opal stop codon (UGA) for tryptophan. The 16S, 23S and 5S rRNA genes are represented as single copies and share the same operon. The manual genome annotation suggests the presence of 1173 CDSs and 31 tRNAs, covering all amino-acids. Putative functions of most of the CDSs are represented as hypothetical proteins (75.62%), which are mostly due to its large repertoire of paralogous genes (63.76%) (Additional file 1: Table S1). These and other genome features were compared with other hemoplasmas and mycoplasmas members of the pneumoniae group (Table 1). The total number of CDSs of *M. haemocanis* classified by role (according to TIGR microbial role categories) was compared to those found in the *M. haemofelis* genome (Table 2).

**Table 1:** **General genomic characteristics of *Mycoplasma haemocanis *strain Illinois compared to members of pneumoniae group of mycoplasmas**

**Characteristic**	**Pneumoniae Group**							
	**Hemoplasmas**				***M. pneumoniae***	***M. gallisepticum***	***M. genitalium***	***M. penetrans***
	***M. haemocanis *strain Illinois**	***M. haemofelis *strain Ohio2**	***'Candidatus *M. haemominutum*' *strain Birmingham 1**	***M. suis *strain Illinois**				
Genome size (base pairs)	919 992	1 155 937	513 880	742 431	816 394	1 012 800	580 076	1 358 633
% of G + C	35.3	38.8	35.5	31.1	40	31	31.7	25.7
Total of genes	1207	1584	582	883	733	817	524	1069
Coding sequences (CDSs)	1173	1549	547	844	689	763	475	1037
CDSs with predicted function	286 (24.3%)	299 (19.3%)	219 (40%)	293 (34.7%)	333 (48.3%)	469 (61.46%)	323 (68%)	585 (56.4%)
No. of tRNAs	31	31	32	32	37	32	36	29
No. of rRNAs								
16S	1	1	1	1	1	2	1	1
23S	1	1	1	1	1	2	1	1
5S	1	1	1	1	1	3	1	1
Genes in paralogous families	748 (63.76%)	1103 (71.2%)	134 (24.5%)	361 (42.8%)	132 (19.1%)	110 (14.4%)	25 (5.2%)	245 (23.6%)

Data was obtained from GenBank database using the following accession numbers: *M. haemocanis* strain Illinois (CP003199.1), *M. haemofelis* strain Ohio2 (CP002808), ‘*Candidatus* Mycoplasma haemominutum’ strain Birmingham 1 (HE613254.1), *M. suis* strain Illinois (CP002525), *M. pneumoniae* (U00089), *M. gallisepticum* (AE015450), *M. genitalium* (L43967), *M. penetrans* (BA000026). Paralogous gene families were assigned using BLASTclust, with 70% coverage and 30% sequence identity thresholds.

**Table 2:** **Comparison of the total number of protein coding sequences (CDSs) of *M. haemocanis *strain Illinois and *M. haemofelis *strain Ohio2 genomes classified by role according to TIGR microbial role categories**

**Role Category**	**Number of CDSs (%)**	
	***M. haemocanis *str. Illinois**	***M. haemofelis *str. Ohio2**
Purines, pyrimidines, nucleosides, and nucleotides	33 (2.81%)	29 (1.85%)
Fatty acid and phospholipid metabolism	6 (0.51%)	6 (0.38%)
Biosynthesis of co-factors, prosthetic groups, and carriers	8 (0.68%)	7 (0.45%)
Central intermediary metabolism	1 (0.09%)	1 (0.06%)
Energy metabolism	25 (2.13%)	22 (1.41%)
Transport and binding proteins	35 (2.98%)	32 (2.04%)
DNA metabolism	44 (3.75%)	52 (3.32%)
Transcription	21 (1.79%)	18 (1.15%)
Protein synthesis	96 (8.18%)	97 (6.2%)
Protein fate	21 (1.79%)	19 (1.21%)
Regulatory functions	4 (0.34%)	3 (0.19%)
Signal transduction	3 (0.26%)	2 (0.13%)
Cell envelope	3 (0.26%)	7 (0.45%)
Cellular processes	14 (1.19%)	10 (0.64%)
Unknown functions	8 (0.68%)	8 (0.51%)
Hypothetical proteins	887 (75.62%)	1253 (80.06%)
Total*	**1209**	**1566**

*Some of the CDSs are in more than one category.

### Metabolic pathways predictions suggest similar growth requirements for *M. haemocanis* and *M. haemofelis*

Prediction of *M. haemocanis* metabolic pathways based on the KEGG pathway database [32] and Yus’s report [33] revealed that they are identical to those predicted for *M. haemofelis*[24]. As shown for *M. haemofelis*, metabolic pathways in *M. haemocanis* are reduced with many of the nutrients and metabolic precursors imported from the blood environment [24]. ATP and DNA/RNA biosynthesis depend on the transport from the environment of glucose and ribose/base derivates, respectively. Imported bases include: hypoxanthine, adenine, guanine, uracil and cytidine 5’-monophosphate (CMP). Furthermore, amino acids, nicotinamide and any vitamins required for growth must be acquired from blood environment.

### Comparative analyses of *M. haemocanis* and *M. haemofelis* genomes

**Figure 1:**
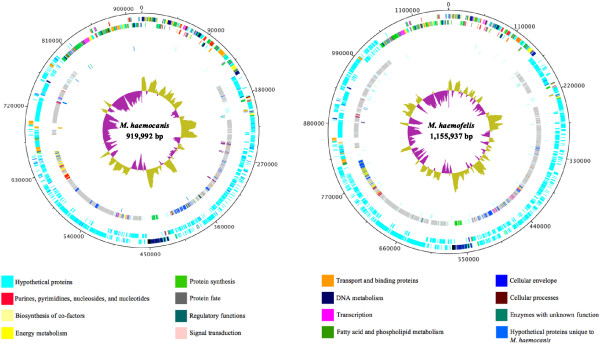
**Circular representation of the genomes of *Mycoplasma haemocanis *strain Illinois and *Mycoplasma haemofelis *strain Ohio2 showing a similar content and organization of the coding sequences.** The *dnaA * gene is at position zero in both genome plots, and the rRNAs (16S, 23S and 5S) are represented in black on the outermost circle. Outer to inner circles correspond to: circle 1: predicted coding sequences (CDSs) on the positive strand; circle 2: predicted CDSs on the negative strand. Each CDS is classified by TIGR role category according to the color designation in the legend below the plots; circle 3: CDSs in paralogous gene families (larger than 5 CDSs) with each family represented by a different color in each genome and homologous families by the same corresponding color in both genomes; circle 4: unique CDSs of each genome with colors corresponding to their role or paralogous family if applicable; circle 5: GC skew. Paralogous families with less than 5 CDSs are represented in light blue. The diagrams were generated using Artemis 12.0 - DNAPlotter version 1.4, Sanger Institute. (*M. haemofelis* plot was modified from Santos et al. [24])

*M. haemocanis* genome was compared in its entirety to *M. haemofelis* strain Ohio2 (Figure 1): *M. haemofelis* has 376 CDSs more than *M. haemocanis*, however the majority of these CDSs are members of paralogous gene families also present in *M. haemocanis*; a set of only 67 CDSs was found to be different between these hemoplasmas. The canine hemoplasma possesses only 20 CDSs not identified in *M. haemofelis* genome, while 47 CDSs were unique to *M. haemofelis*. Most of these CDSs are hypothetical proteins, including one family of paralogous genes from *M. haemocanis* and four paralogous gene families from *M. haemofelis* (Additional file 2: Table S2). Predicted functions based on protein sequence similarity for these particular sets of CDSs were assigned using ESG software [36] (Additional file 2: Table S2). Analyses based on PSORTb parameters show that 35% of the unique CDSs of *M. haemocanis* are associated with the cytoplasmic membrane, while 17% of *M. haemofelis* CDSs are predicted to be associated with the membrane and 6.4% with extracellular (signal peptide detected) localization. For most of the unique CDSs, an unknown subcellular localization was predicted, corresponding to 60% and 51% in *M. haemocanis* and *M. haemofelis* genomes, respectively (Table 3). Thirteen out of the 20 (65%) unique CDSs of *M. haemocanis* and 35 out of 47 (74.5%) of *M. haemofelis* have at least one internal helix predicted. The number of predicted helices is also shown in the Additional file 2: Table S2. Fifteen lipoproteins were predicted in *M. haemocanis* compared with 17 for *M. haemofelis* genome, 13 of them are conserved between the two species. In addition, we identified 33 variable number tandem repeats (VNTRs) in the genome of *M. haemocanis* genome (Additional file 3: Table S3), while 61 were reported for *M. haemofelis*[24]. As in *M. haemofelis* genome, most of the VNTRs of *M. haemocanis* were localized within intergenic regions of hypothetical proteins. Five VNTRs were identified within the Type I restriction system operon; the presence of VNTRs in this operon was also described for *M. haemofelis*[24]. Other *M. haemocanis* VNTRs were identified within CDSs for SecD protein, efflux ABC transporter permease protein, PtsG protein, enolase, PotD protein, and for some of the hypothetical proteins.

**Table 3:** **Subcellular localization of the unique protein coding sequences (CDSs) of *M. haemocanis *strain Illinois and *M. haemofelis *strain Ohio2 genomes**

**Subcellular Localization**^**a**^	Number of unique CDSs*					
	***M. haemocanis *strain Illinois**			***M. haemofelis *strain Ohio2**		
	**CDSs in paralogous families**	**CDSs not in paralogous families**	**CDSs with predicted function**	**CDSs in paralogous families**	**CDSs not in paralogous families**	**CDSs with predicted function**
Cytoplasmic membrane	3	3	1	0	8	0
Cytoplasmic	0	0	1	3	7	2
Extracellular	0	0	0	3	0	0
Unknown	5	6	1	18	5	1
**Total**	**8**	**9**	**3**	**24**	**20**	**3**

^**a**^ prediction using PSORTb version 3 software.

* Unique CDSs were assigned using BLASTp when there were no matching sequences with ≥ 90% coverage and ≥ 30% identity or ≥ 80% coverage and ≥ 40% identity to the query sequence.

Only 3 CDSs with known function are exclusive to *M. haemocanis* genome when compared to *M. haemofelis* strain Ohio2; however phosphotransferase system glucose-specific IIBC component (MHC_04460) is only present in the genome of *M. haemofelis* strain Langford, while two ribosomal proteins (MHC_00995 and MHC_05355) are in neither of the feline hemoplasma strains (Additional file 2: Table S2). Another 3 CDSs with known function were identified only in the genome of *M. haemofelis*: two of these proteins are C-5 cytosine-specific DNA methylases (MHF_1273 and MHF_1319), and the other protein is a type II site-specific deoxyribonuclease (MHF_1274) (Additional file 2: Table S2). Small CDSs (corresponding to 30–100 amino acids) characterized as fragments of paralogous genes were excluded from these analyses since they presented a coverage and/or identity below the cutoff to be considered as a member of a paralogous gene family (70% coverage and 30% identity threshold).

### Phylogenomic comparison of *M. haemocanis* to other hemoplasmas

ANI and tetranucleotide signature correlation indexes are shown in Table 4. As indicated, *M. haemocanis* had an ANI of approximately 85% in comparison to all other hemoplasma genomes, including *M. haemofelis*. This is below the cutoff value of 94% for species circumscription. The tetranucleotide correlation indexes of *M. haemocanis* with other genomes were also below the 0.99 cutoff limit, being approximately 0.95 for *M. haemofelis* strains and 0.45 for *M. suis* strains. Based on these analyses, *M. haemocanis* is indeed a distinct species infecting the dog.

**Table 4:** Average nucleotide identity* (ANI) and tetranucleotides signature correlation indexes (Tetra) of selected hemotrophic mycoplasmas

	***M. haemofelis *Ohio2**		***M. haemofelis *Langford**		***M. suis *illinois**		***M. suis *KI3806**	
	**ANI**	**Tetra**	**ANI**	**Tetra**	**ANI**	**Tetra**	**ANI**	**Tetra**
***M. haemocanis *Illinois**	85.11	0.959	85.21	0.962	85.59	0.452	85.5	0.453
***M. haemofelis *Ohio2**			97.3	0.999	85.41	0.365	85.3	0.372
***M. haemofelis *Langford**					84.83	0.366	87.74	0.372
***M. suis *illinois**							97.74	0.997

* = ANI was calculated using MUMmer algorithm in JSpecies software.

As expected, strains of the same species (*M. suis* Illinois and KI3806; *M. haemofelis* Ohio2 and Langford1) showed high ANI and tetranucleotide correlation indexes, which were above the proposed thresholds for species definition. In contrast, ANI and tetranucleotide correlation indexes between *M. suis* and *M. haemofelis* were approximately 85% and 0.37, respectively, correctly separating these organisms as two different species of mycoplasmas.

## Discussion

The complete genome sequence and annotation of *M. haemocanis* extends our understanding of the biology of hemoplasmas and provides clues about the growth requirements for *in vitro* cultivation of these bacteria. Based on the metabolic pathway predictions and specific metabolic deficiencies, a more comprehensive medium can be designed [33]. To date, only three other species of hemoplasmas have been entirely sequenced [23-27]. The genome features of *M. haemocanis*, including its small size, low G + C content and use of UGA codon to encode tryptophan, are similar to those of other hemoplasmas and are typical of members of the genus *Mycoplasma*. It is believed that the reduced metabolic pathways of hemoplasmas are probably a consequence of the adaptation to the nutrient-rich blood environment [23,24]. The predicted metabolic pathways of *M. haemocanis* are very similar to those of *M. haemofelis* having orthologs for all the CDSs identified in the genome of this feline hemoplasma [24]; this is not surprising since both species are obligate red cell pathogens that reside in the blood of their hosts. As suggested for other hemoplasmas, it is likely that *M. haemocanis* takes advantage of the erythrocyte’s metabolism, scavenging nutrients, which leads to diminished erythrocyte life-span and exacerbation of anemia during acute disease.

Additional primary virulence factors were not identified in the genome of *M. haemocanis*. The *o*-sialoglycoprotein endopeptidase, related to the cleavage of glycophorin A, is conserved among hemoplasmas; the superoxide dismutase (SOD), identified in *M. haemofelis*[24,26] is also present in *M. haemocanis*, but not found in any other sequenced mycoplasma. Although SOD may protect these bacteria from superoxide anion toxicity faced in the blood environment, it is unlikely that this enzyme plays a determinant role in the primary pathogenicity associated with *M. haemofelis* infection or in the opportunistic infection caused by *M. haemocanis*.

As with other hemoplasmas, *M. haemocanis* contains an abundance of paralogous gene families (63.7% of all its CDSs) and the presence of strategically located tandem repeats. Although there is evidence supporting the role of paralog genes and the presence of tandem repeats in the development of antigenic diversity in *Mycoplasma* species [38,39], additional studies are needed to verify the ability of hemoplasmas to undergo antigenic variation. The presence of irregular cyclic episodes of bacteremia in splenectomized dogs reported following experimental infection with *M. haemocanis*[40], and the possibility that such cycles are due to phase variation is also an area of active investigation in our laboratory.

Comparison of the genomes of *M. haemocanis* and *M. haemofelis* revealed remarkable genetic similarities. Most of the coding and non-coding sequences were conserved and topography of genes within their chromosomes was similar. Even the paralogous gene families were conserved between the two species; the only exceptions were one family with 8 members in *M. haemocanis*, and four small families of *M. haemofelis* with 8, 5, 4 and 3 members, and two with 2 members. The major difference in the paralogous families is the number of duplicate genes inside each of the common families. Thus, as with other bacteria that cannot survive without their host, it appears that maintaining paralogous gene families to generate antigenic variants is a high priority for the hemoplasmas too [41]. On the other hand, CDSs that are unique to *M. haemocanis* or *M. haemofelis* might represent a set of proteins related to differences in virulence and/or related to host specificity. Most of these unique proteins are hypothetical. Although we attempted to improve the function prediction accuracy using the ESG software [36], most of the probabilities assigned were less than 50% and results remained inconclusive (Additional file 2: Table S2). Regarding the subcellular localization of the unique CDSs, it is important to mention that the PSORTb software only predicts cytoplasmic membrane localizations when 3 or more transmembrane helices are present within the sequence, otherwise unknown localization is returned. Therefore, these predictions based on strict criteria might have underestimated the potential for membrane localization of these CDSs.

CDSs with known function that are unique to *M. haemocanis* do not appear to have a significant impact on its pathogenicity since they code for an enzyme involved in sugar transport and for ribosomal proteins. On the other hand, *M. haemofelis* possesses a type II restriction enzyme and two C-5 cytosine-specific DNA methylases (C5 Mtase); the restriction endonuclease is located in the same operon as one of the C5 Mtase, indicating that this operon is functional [42]. Moreover, this endonuclease/methyltranferase pair is not present in any of the other hemoplasmas and the restriction enzyme is absent in the strain Langford 1 of *M. haemofelis*. DNA methylation has been associated with virulence in other bacteria [43]; however, the function of these pair in *M. haemofelis* Ohio2 is unknown.

As mentioned previously, the hemoplasmas cannot be cultivated *in vitro*. This has resulted in a lack of detailed phenotypic and genotypic characterization, which has hampered our ability to correctly classify these organisms within the *Mycoplasmataceae* family. In addition, the 16S rRNA gene failed to provide sufficient resolution to separate *M. haemocanis* and *M. haemofelis* as different species of *Mycoplasma*[5,17]. To date, the genotypic evidence for species differentiation of these two hemoplasmas is solely based on phylogenetic studies using a 177 bp fragment of their RNase P genes [18,44]. Herein, we performed a phylogenomic comparison between *M. haemocanis* and strains of *M. haemofelis* to resolve this long lasting controversy. In recent years, the sequencing of entire genomes has allowed the in silico evaluation of genomic similarities between different organisms. ANI and tetranucleotide signatures have been used as surrogates to previous methods of species circumscription, such as 16S rRNA gene phylogeny and DNA-DNA hybridization [37]. With both ANI and tetranucleotide indexes below the proposed thresholds for species definition, our results show that the *M. haemocanis* strain Illinois and *M. haemofelis* (strains Langford and Ohio2) are different species of mycoplasmas infecting two distinct animal species. This conclusion is also supported by the transmission studies done more than 50 years ago [8].

Taken together our results suggest that, although sharing very similar genomes, *M. haemocanis* and *M. haemofelis* are different mycoplasmal species infecting dogs and cats, respectively. The set of unique proteins may be a target for vaccine development against these hemoplasmas, especially for the feline hemoplasmosis that can cause acute disease in immunocompetent hosts.

### Nucleotide sequence accession number

The genome of *M. haemocanis* strain Illinois was deposited in GenBank under the accession number CP003199.1.

## Competing interests

The authors declare that they have no competing interests.

## Authors’ contributions

Conceived and designed experiments: NCN, APS, AMSG, JBM. Performed experiments: NCN, APS, AMSG, JBM. Analyzed the data: NCN, APS, AMSG, PJS, JBM. Wrote and reviewed the paper: NCN, APS, AMSG, PJS, JBM. All authors read and approved the final manuscript.

## Supplementary Material

Additional file 1 **Table S1.** List of paralogous gene families* in the genome of *Mycoplasma haemocanis* strain Illinois. Complete list of paralogous gene families in *Mycoplasma haemocanis* strain Illinois genome assigned by BLASTclust tool provided by Max-Planck Institute for Developmental Biology, with 70% covered length and 30% sequence identity thresholds. GenBank accession numbers are provided. Click here for file

Additional file 2 **Table S2.** Analyses of the unique protein coding sequences (CDSs) of *Mycoplasma haemocanis* strain Illinois and *M. haemofelis* strain Ohio2. Analyses of all the unique CDSs of *M. haemocanis* strain Illinois and *M. haemofelis* strain Ohio2 were performed using ESG software, which predicts protein functions based on sequence similarity, and PSORTb version 3 software, which predicts subcellular localization. GenBank accession numbers of proteins corresponding to each CDS are also provided. Click here for file

Additional file 3 **Table S3.** Tandem repeats* identified in the genome of *Mycoplasma haemocanis* strain Illinois. Complete list of variable tandem repeats, identified using Tandem repeats finder program, indicating their sequences and position in the genome of *M. haemocanis.* Click here for file
