# Supplementary material for: Mycoplasma haemocanis – the canine hemoplasma and its feline counterpart in the genomic era
Source: Vet Res. 2012 Sep 28;43(1):66. doi: 10.1186/1297-9716-43-66 (PMC3496576; doi:10.1186/1297-9716-43-66)
Supplement: Additional file 1 — Table S1. List of paralogous gene families* in the genome of Mycoplasma haemocanis strain Illinois. Complete list of paralogous gene families in Mycoplasma haemocanis strain Illinois genome assigned by BLASTclust tool provided by Max-Planck Institute for Developmental Biology, with 70% covered length and 30% sequence identity thresholds. GenBank accession numbers are provided. [file 1297-9716-43-66-S1.pdf]

Supplementary Table 1. List of paralogous gene families* in the genome of *Mycoplasma haemocanis* strain Illinois.

Family 1, 514 genes

AEW45559.2
AEW45549.1
AEW45199.2
AEW45473.1
AEW45478.1
AEW45444.1
AEW45834.1
AEW45488.1
AEW45805.1
AEW45112.1
AEW45744.1
AEW45156.1
AEW45438.1
AEW45441.1
AEW45547.1
AEW45111.2
AEW45137.1
AEW45202.2
AEW45116.1
AEW45120.1
AEW45129.1
AEW45198.1
AEW45443.1
AEW45735.2
AEW45788.1
AEW45073.2
AEW45079.1
AEW45126.1
AEW45170.1
AEW45439.1
AEW45530.1
AEW45094.1
AEW45098.1
AEW45114.1
AEW45117.1
AEW45131.1
AEW45132.1
AEW45588.2
AEW45610.1
AEW45762.1
AEW45768.1
AEW45783.1
AEW45164.1
AEW45577.1
AEW45736.1
AEW45742.1
AEW45121.1
AEW45177.1

AEW45734.1
AEW45840.1
AEW45091.1
AEW45118.1
AEW45434.1
AEW45741.1
AEW45074.1
AEW45081.1
AEW45082.1
AEW45088.1
AEW45110.2
AEW45809.1
AEW45075.1
AEW45089.1
AEW45127.1
AEW45210.1
AEW45496.2
AEW45543.2
AEW45583.1
AEW45615.1
AEW45837.1
AEW45860.1
AEW45077.1
AEW45113.1
AEW45135.1
AEW45200.1
AEW45455.1
AEW45858.1
AEW45096.1
AEW45097.1
AEW45099.1
AEW45147.1
AEW45165.1
AEW45784.1
AEW45807.1
AEW45019.1
AEW45076.1
AEW45080.1
AEW45125.1
AEW45437.1
AEW45534.1
AEW45584.1
AEW45743.1
AEW45093.1
AEW45138.1
AEW45157.1
AEW45167.1
AEW45302.1
AEW45737.1
AEW45740.1
AEW45769.1
AEW45794.1
AEW45100.1
AEW45176.1
AEW45188.1

AEW45435.2
AEW45506.1
AEW45789.1
AEW45856.1
AEW45012.1
AEW45020.1
AEW45078.1
AEW45141.2
AEW45515.1
AEW45827.1
AEW45033.1
AEW45084.1
AEW45152.1
AEW45236.1
AEW45285.1
AEW45363.1
AEW45787.1
AEW45797.1
AEW45851.1
AEW45876.1
AEW45009.1
AEW45027.1
AEW45211.1
AEW45213.1
AEW45216.1
AEW45242.1
AEW45522.1
AEW45590.1
AEW45763.1
AEW45774.2
AEW45780.1
AEW45798.1
AEW45830.1
AEW45853.1
AEW45855.1
AEW45021.1
AEW45032.2
AEW45034.1
AEW45036.1
AEW45139.1
AEW45149.1
AEW45160.1
AEW45185.1
AEW45205.1
AEW45231.1
AEW45243.1
AEW45249.1
AEW45261.2
AEW45281.1
AEW45348.1
AEW45535.1
AEW45680.1
AEW45839.1
AEW45857.1
AEW45015.1

AEW45024.1
AEW45035.2
AEW45038.1
AEW45083.1
AEW45128.1
AEW45192.1
AEW45240.1
AEW45255.1
AEW45260.1
AEW45268.1
AEW45299.1
AEW45300.1
AEW45354.1
AEW45433.2
AEW45442.1
AEW45495.2
AEW45544.1
AEW45582.2
AEW45759.1
AEW45852.1
AEW45018.1
AEW45092.1
AEW45140.1
AEW45182.2
AEW45214.1
AEW45219.1
AEW45247.1
AEW45304.1
AEW45492.1
AEW45497.1
AEW45524.1
AEW45565.1
AEW45689.1
AEW45791.1
AEW45835.1
AEW45872.1
AEW45014.1
AEW45023.1
AEW45194.1
AEW45195.1
AEW45218.1
AEW45226.1
AEW45276.1
AEW45428.1
AEW45512.1
AEW45568.1
AEW45585.1
AEW45597.1
AEW45785.1
AEW45819.1
AEW45826.1
AEW45028.1
AEW45104.1
AEW45105.1
AEW45106.1

AEW45146.1
AEW45186.1
AEW45208.1
AEW45230.1
AEW45504.1
AEW45511.1
AEW45563.2
AEW45570.1
AEW45579.1
AEW45591.1
AEW45658.1
AEW45661.2
AEW45795.1
AEW45800.1
AEW45011.1
AEW45122.1
AEW45220.1
AEW45234.1
AEW45250.1
AEW45352.1
AEW45454.1
AEW45502.1
AEW45508.2
AEW45532.2
AEW45552.1
AEW45592.2
AEW45777.1
AEW45793.1
AEW45838.1
AEW45039.1
AEW45102.1
AEW45119.1
AEW45161.2
AEW45221.1
AEW45239.1
AEW45313.1
AEW45483.1
AEW45519.1
AEW45587.1
AEW45612.2
AEW45761.1
AEW45771.1
AEW45775.1
AEW45778.2
AEW45842.1
AEW45004.1
AEW45168.1
AEW45204.1
AEW45223.1
AEW45229.1
AEW45246.1
AEW45248.1
AEW45267.1
AEW45287.1
AEW45305.1

AEW45332.1
AEW45333.1
AEW45353.1
AEW45359.2
AEW45523.1
AEW45545.1
AEW45554.1
AEW45578.1
AEW45596.1
AEW45667.1
AEW45767.1
AEW45773.1
AEW45782.1
AEW45806.1
AEW45859.1
AEW45890.1
AEW45143.1
AEW45145.1
AEW45189.1
AEW45207.1
AEW45217.2
AEW45253.2
AEW45254.1
AEW45256.1
AEW45289.1
AEW45298.1
AEW45301.1
AEW45362.2
AEW45493.1
AEW45517.1
AEW45531.1
AEW45562.1
AEW45573.1
AEW45602.1
AEW45616.1
AEW45687.1
AEW45810.2
AEW45854.1
AEW45885.1
AEW45022.1
AEW45115.1
AEW45159.1
AEW45180.1
AEW45212.1
AEW45241.1
AEW45269.2
AEW45283.1
AEW45326.1
AEW45452.1
AEW45479.1
AEW45560.1
AEW45572.1
AEW45593.1
AEW45598.1
AEW45611.1

AEW45628.1
AEW45684.1
AEW45765.1
AEW45829.1
AEW45849.1
AEW45877.1
AEW45003.2
AEW45190.1
AEW45197.2
AEW45232.1
AEW45244.1
AEW45271.1
AEW45472.1
AEW45550.1
AEW45569.1
AEW45595.1
AEW45601.1
AEW45627.1
AEW45770.1
AEW45882.1
AEW45889.1
AEW45103.1
AEW45178.1
AEW45224.1
AEW45252.1
AEW45257.1
AEW45259.1
AEW45286.2
AEW45318.1
AEW45429.1
AEW45445.1
AEW45490.1
AEW45513.1
AEW45516.1
AEW45520.1
AEW45527.1
AEW45546.2
AEW45555.1
AEW45613.1
AEW45655.2
AEW45656.1
AEW45657.1
AEW45678.1
AEW45801.2
AEW45868.1
AEW45871.1
AEW45144.1
AEW45163.1
AEW45187.1
AEW45319.1
AEW45427.1
AEW45440.1
AEW45471.1
AEW45499.1
AEW45528.1

AEW45561.1
AEW45567.1
AEW45571.1
AEW45574.1
AEW45580.1
AEW45600.1
AEW45609.1
AEW45617.1
AEW45008.1
AEW45148.1
AEW45153.1
AEW45172.1
AEW45175.1
AEW45225.1
AEW45436.1
AEW45505.1
AEW45510.1
AEW45514.1
AEW45521.1
AEW45529.1
AEW45553.2
AEW45566.1
AEW45589.1
AEW45623.1
AEW45631.1
AEW45634.1
AEW45688.1
AEW45764.1
AEW45772.1
AEW45790.1
AEW45844.1
AEW45883.1
AEW45884.1
AEW45030.1
AEW45171.1
AEW45196.1
AEW45321.1
AEW45355.2
AEW45426.1
AEW45453.1
AEW45501.1
AEW45518.1
AEW45526.1
AEW45576.1
AEW45618.1
AEW45668.1
AEW45766.1
AEW45776.1
AEW45850.1
AEW45865.1
AEW45867.1
AEW45880.1
AEW45010.1
AEW45046.1
AEW45150.1

AEW45151.1
AEW45277.1
AEW45278.1
AEW45303.1
AEW45451.1
AEW45491.1
AEW45525.1
AEW45564.1
AEW45586.1
AEW45594.1
AEW45599.1
AEW45760.1
AEW45786.1
AEW45792.1
AEW45825.1
AEW45154.1
AEW45290.1
AEW45475.1
AEW45548.1
AEW45659.1
AEW45679.1
AEW45779.1
AEW45881.1
AEW45888.1
AEW45201.1
AEW45258.1
AEW45476.1
AEW45494.1
AEW45660.1
AEW45686.1
AEW45173.1
AEW45803.2
AEW45280.1
AEW45292.2
AEW45329.1
AEW45558.2
AEW45832.1
AEW45507.1
AEW45575.1
AEW45635.1
AEW45796.1
AEW45886.1
AEW45017.1
AEW45270.1
AEW45320.1
AEW45328.1
AEW45509.1
AEW45044.1
AEW45006.1
AEW45041.1
AEW45215.1
AEW45311.1
AEW45498.1
AEW45025.1
AEW45758.1

AEW45013.1
AEW45685.1
AEW45820.1
AEW45288.1
AEW45005.1
AEW45016.1
AEW45040.1
AEW45861.1
AEW45828.1
AEW45804.1
AEW45781.1
AEW45808.1
AEW45836.1
AEW45799.1
AEW45833.1
AEW45831.1
AEW45841.2
AEW45581.1
AEW45533.1
AEW45614.1
AEW45090.1
AEW45087.1
AEW45551.1
AEW45843.1
AEW45500.1
AEW45085.1

Family 2, 53 genes

AEW45340.1
AEW45557.1
AEW45360.1
AEW45365.2
AEW45537.1
AEW45539.1
AEW45541.1
AEW45337.1
AEW45339.2
AEW45364.1
AEW45367.2
AEW45338.1
AEW45536.1
AEW45556.1
AEW45341.1
AEW45538.1
AEW45357.2
AEW45540.1
AEW45347.2
AEW45484.1
AEW45343.1
AEW45263.1
AEW45361.2
AEW45227.1
AEW45606.1
AEW45351.2

AEW45358.2
AEW45604.1
AEW45349.1
AEW45265.1
AEW45608.1
AEW45356.1
AEW45605.1
AEW45264.1
AEW45345.1
AEW45603.1
AEW45262.1
AEW45266.1
AEW45350.1
AEW45191.1
AEW45206.1
AEW45342.1
AEW45209.1
AEW45346.1
AEW45228.1
AEW45607.2
AEW45203.1
AEW45179.1
AEW45312.2
AEW45322.1
AEW45316.1
AEW45181.1
AEW45183.1

Family 3, 25 genes

AEW45664.1
AEW45697.1
AEW45706.1
AEW45459.1
AEW45654.1
AEW45465.1
AEW45690.1
AEW45462.1
AEW45677.1
AEW45693.1
AEW45675.1
AEW45431.2
AEW45666.1
AEW45670.1
AEW45486.1
AEW45701.1
AEW45310.2
AEW45708.1
AEW45461.2
AEW45702.2
AEW45665.1
AEW45705.1
AEW45676.2
AEW45682.1
AEW45683.1

Family 4, 18 genes

AEW45644.2
AEW45643.1
AEW45640.1
AEW45481.1
AEW45639.1
AEW45646.1
AEW45845.2
AEW45648.1
AEW45645.1
AEW45641.1
AEW45647.1
AEW45673.1
AEW45811.1
AEW45649.1
AEW45813.1
AEW45662.2
AEW45671.1
AFM45085.1

Family 5, 18 genes

AEW45029.1
AEW45814.2
AEW45458.1
AEW45470.1
AEW45691.1
AEW45707.1
AEW45456.1
AEW45669.1
AEW45695.1
AEW45824.1
AEW45822.1
AEW45674.1
AEW45704.1
AEW45466.1
AEW45698.2
AEW45468.1
AEW45696.1
AEW45703.1

Family 6, 14 genes

AEW45413.2
AEW45405.1
AEW45407.1
AEW45408.1
AEW45409.1
AEW45415.1
AEW45403.1
AEW45411.1
AEW45402.1
AEW45410.1

AEW45401.1
AEW45414.1
AEW45412.1
AEW45404.2

Family 7, 12 genes

AEW45626.2
AEW45642.1
AEW45625.1
AEW45636.1
AEW45622.1
AEW45633.1
AEW45632.1
AEW45629.1
AEW45630.1
AEW45621.1
AEW45620.1
AFM45088.1

Family 8, 9 genes

AEW45377.2
AEW45480.1
AEW45460.1
AEW45457.1
AEW45672.1
AEW45482.1
AEW45823.1
AEW45694.1
AEW45812.1

Family 9, 8 genes

AEW45136.1
AEW45133.2
AEW45130.1
AEW45134.1
AEW45237.1
AEW45155.1
AEW45158.1
AEW45238.1

Family 10, 6 genes

AEW45372.2
AEW45334.1
AEW45330.1
AEW45293.1
AEW45732.1
AEW45370.2

Family 11, 5 genes

AEW45331.1

AEW45373.1
AEW45294.1
AEW45295.1
AFM45077.1

Family 12, 5 genes

AEW45284.2
AEW45222.1
AEW45142.1
AEW45251.2
AEW45376.1

Family 13, 5 genes

AEW45485.1
AEW45430.1
AEW45327.1
AEW45325.2
AEW45282.1

Family 14, 4 genes

AEW45450.2
AEW45423.1
AEW45425.1
AEW45449.2

Family 15, 4 genes

AEW45879.1
AEW45887.1
AEW45862.1
AEW45864.1

Family 16, 4 genes

AEW45042.1
AEW45026.1
AEW45007.1
AEW45045.1

Family 17, 4 genes

AEW45446.1
AEW45619.1
AEW45273.1
AEW45624.1

Family 18, 4 genes

AEW45166.1
AEW45169.1
AEW45162.2
AEW45681.1

Family 19, 3 genes

AEW45057.1
AEW45058.2
AFM45072.1

Family 20, 3 genes

AEW45869.1
AEW45866.1
AEW45002.1

Family 21, 3 genes

AEW45174.1
AEW45272.1
AEW45123.1

Family 22, 3 genes

AEW45317.1
AEW45464.1
AEW45692.1

Families of 2 gene members (12 families)

AEW45385.1
AEW45384.1

AEW45873.1
AEW45874.2

AEW45417.1
AEW45406.2

AEW44918.1
AEW44919.1

AEW45421.1
AEW45369.1

AEW44969.1
AEW44968.1

AEW45487.1
AEW45309.1

AEW45821.1
AEW45818.1

AEW45031.1
AFM45091.1

AEW45296.1

AEW45374.1

AEW45738.1
AEW45733.1

AEW45314.1
AEW45324.1

---

*The assignment of paralogous gene families was performed using BLASTclust tool provided by Max-Planck Institute for Developmental Biology, with 70% covered length and 30% sequence identity thresholds.

GenBank accession numbers are provided.
