# Supplementary material for: Mycoplasma haemocanis – the canine hemoplasma and its feline counterpart in the genomic era
Source: Vet Res. 2012 Sep 28;43(1):66. doi: 10.1186/1297-9716-43-66 (PMC3496576; doi:10.1186/1297-9716-43-66)
Supplement: Additional file 2 — Table S2. Analyses of the unique protein coding sequences (CDSs) of Mycoplasma haemocanis strain Illinois and M. haemofelis strain Ohio2. Analyses of all the unique CDSs of M. haemocanis strain Illinois and M. haemofelis strain Ohio2 were performed using ESG software, which predicts protein functions based on sequence similarity, and PSORTb version 3 software, which predicts subcellular localization. GenBank accession numbers of proteins corresponding to each CDS are also provided. [file 1297-9716-43-66-S2.pdf]

Supplementary Table 2. Analyses of the unique protein coding sequences (CDSs) of *Mycoplasma haemocanis* strain Illinois and *M. haemofelis* strain Ohio2.

M. haemocanis str. Illinois unique CDSs	Role category (TIGR microbial role categories)	Molecular Function Terms[a]	Biological Process Terms[a]	Cellular Component Terms[a]	Internal Helix[b]	Non-Cytoplasmic (signal peptide)[b]	Subcellular Localization[b]	GenBank Accession Number
Paralogous family 9	hypothetical protein	ion/voltage-gated chloride channel activity (41%)	ion/chloride transport (41%)	membrane (42%)	1	no	cytoplasmic membrane	AEW45130.1
	hypothetical protein	catalytic activity (35%)	metabolic process (35%)	cytoplasm (21%)	1	no	unknown	AEW45133.1
	hypothetical protein	transferase (33%)	transport (30%)	cytoplasm (53%)	1	no	cytoplasmic membrane	AEW45134.1
	hypothetical protein	nucleotide/ATP binding (39%)	metabolic process (32%)	cytoplasm (37%)	1	no	unknown	AEW45136.1
	hypothetical protein	catalytic activity (51%)	GTP metabolic process (32%)	cytoplasm (67%)	1	no	unknown	AEW45155.1
	hypothetical protein	ATP binding (39%)	carbohydrate metabolic process (37%)	cytoplasm (28%)	1	no	unknown	AEW45158.1
	hypothetical protein	nucleotide binding (37%)	metabolic process (24%)	ribosome (32%)/cytoplasm (24%)	1	no	cytoplasmic membrane	AEW45237.1
	hypothetical protein	electron carrier activity (38%)/oxidoreductase activity (38%0	fumarate metabolic process (22%)	membrane (46%)	1	no	unknown	AEW45238.1
Not in paralogous families	hypothetical protein	ATP binding (39%)	cell adhesion (19%)	cytoplasm (20%)/extracellular (19%)	0	no	cytoplasmic membrane	AEW45062.1
	hypothetical protein	protein binding (46%)/ two-component response regulator activity (46%)	two-component signal transduction system (46%)/ regulation of transcription, DNA-dependent (46%)/ response to hydrogen peroxide (46%)	membrane (53%)	0	no	unknown	AEW45474.1
	hypothetical protein	nucleotide/ATP binding (100%)	DNA metabolic process/DNA topological change (100%)	chromosome (100%)	2	no	cytoplasmic membrane	AEW45815.1
	hypothetical protein	ATP binding (37%)	ATP biosynthetic process (22%)	membrane (70%)	1	no	cytoplasmic membrane	AEW45816.1
	hypothetical protein	hydrolase (71%)	polysaccharide catabolic process (71%)	membrane (71%)	1	no	unknown	AEW45817.1
	hypothetical protein	oxidoreductase activity/catalytic activity (42%)	transport (53%)	membrane (53%)	1	no	unknown	AEW45917.1
	hypothetical protein	methyltransferase (68%)	rRNA modification (37%)	cytoplasm (30%)	0	no	unknown	AEW46011.1
	hypothetical protein	N.A.*	N.A.*	N.A.*	0	no	unknown	AEW453231.1
	hypothetical protein	N.A.*	N.A.*	N.A.*	0	no	unknown	AEW45878.1|
CDSs with predicted function (present in other bacteria species, but absent in *M. haemofelis* str. Ohio2)	50S ribosomal protein L33	structural constituent of ribosome (100%)	translation (100%)	ribosome (100%)	0	no	unknown	AEW45065.1
	phosphotransferase system glucose-specific IIBC component	sugar:hydrogen symporter activity (100%)	transport (100%)	membrane (100%)	7	no	cytoplasmic membrane	AEW45748.1
	30S ribosomal protein S14 type Z	structural constituent of ribosome (100%)	translation (100%)	ribosome (100%)	0	no	cytoplasmic	AEW45925.1

M. haemofelis str. Ohio2 unique CDSs	Role category (TIGR microbial role categories)	Molecular Function Terms[a]	Biological Process Terms[a]	Cellular Component Terms[a]	Internal Helix[b]	Non-Cytoplasmic (signal peptide)[b]	Subcellular Localization[b]	GenBank Accession Number
Paralogous family 8	hypothetical protein	metal ion binding/DNA binding (80%)	DNA integration (80%)	N.I.*	1	no	unknown	AEG72490.1
	hypothetical protein	hydrolase (20%)	lipid metabolism (20%)	membrane (42%)	1	no	cytoplasmic	AEG72498.1
	hypothetical protein	signal transducer activity/ receptor activity (54%)	signal transduction (54%)	membrane (56%)	1	no	unknown	AEG72503.1
	hypothetical protein	transferase (19%)	transport (18%)	membrane (35%)	1	yes	extracellular	AEG73680.1
	hypothetical protein	nucleic acid binding (19%)	biological process (13%)	membrane (40%)	1	no	cytoplasmic	AEG72504.1
	hypothetical protein	N.A.*	transport (91%)	chromosome (91%)	1	no	cytoplasmic	AEG73619.1
	hypothetical protein	nucleic acid binding (39%)/ hydrolase (39%)	mRNA processing (39%)	chromosome (72%)	1	no	unknown	AEG73558.1
	hypothetical protein	electron carrier activity (28%)/ heme binding (28%)	transport (28%)	membrane (24%)	1	no	unknown	AEG73559.1
Paralogous family 11	hypothetical protein	catalytic activity (36%)/ metal ion binding (36%)	cell adhesion (23%)	membrane (27%)	1	no	unknown	AEG72489.1
	hypothetical protein	nucleic acid binding (41%)/ hydrolase (41%)	DNA repair (37%)	chromosome (52%)	1	no	unknown	AEG72502.1
	hypothetical protein	heat shock protein binding (49%)	protein transport across membrane (49%)	membrane (41%)	1	yes	unknown	AEG72495.1
	hypothetical protein	ion/voltage-gated chloride channel activity (35%)	transport (45%)	membrane (53%)	1	yes	unknown	AEG72501.1
	hypothetical protein	protein binding (25%)	protein transport (38%)	cytoplasm (38%)	1	yes	unknown	AEG72499.1
Paralogous family 12	hypothetical protein	protein binding (42%)	protein localization (26%)	cytoplasm (56%)	1	yes	unknown	AEG73634.1
	hypothetical protein	nucleotide/ATP binding (45%)	translation (20%)	cytoplasm (27%)	1	yes	extracellular	AEG73653.1
	hypothetical protein	nucleic acid binding (29%)	DNA catabolic process (15%)	membrane (28%)/ cytoplasm (27%)	1	yes	unknown	AEG73621.1
	hypothetical protein	catalytic activity (32%)/ DNA binding (32%)	regulation of transcription (22%)/ DNA recombination/integration (20%)	chromosome (43%)	0	no	unknown	AEG73589.1
Paralogous family 6.3	hypothetical protein	DNA binding (37%)	regulation of transcription (34%)	chromosome (34%)	0	yes	unknown	AEG73145.1
	hypothetical protein	metal ion binding (55%)	transcription (26%)	cytoplasm (40%)	0	no	unknown	AEG73146.1
	hypothetical protein	DNA binding (37%)	regulation of transcription (37%) and replication (35%)	host cell nucleus (35%)/ chromosome (34%)	1	no	unknown	AEG73144.1
Paralogous family 14.2	hypothetical protein	RNA binding (74%)	RNA processing (64%)	cytoplasm (25%)	1	no	unknown	AEG73227.1
	hypothetical protein	nucleotide/ATP binding (27%)	transport (26%)	membrane (59%)	1	no	unknown	AEG73223.1
Paralogous family 15.2	hypothetical protein	transferase (58%)	spermidine catabolic process (34%)	membrane (29%)	1	no	unknown	AEG72719.1
	hypothetical protein	metal ion binding (56%)	protein amino acid phosphorylation (28%)	cytoplasm (50%)	1	no	unknown	AEG72717.1
Not in paralogous families	hypothetical protein	DNA binding (38%)	DNA repair/recombination (37%)	intracellular (44%)	0	no	cytoplasmic membrane	AEG72934.1
	hypothetical protein	hydrolase (30%)/ protein binding (22%)	regulation of transcription (21%)/ pathogenesis (10%)	chromosome (31%)/ membrane (22%)	1	yes	unknown	AEG72464.1
	hypothetical protein	nucleotide binding (43%)	regulation of transcription (34%)	membrane (42%)/ chromosome (28%)	0	no	cytoplasmic	AEG73312.1
	hypothetical protein	catalytic activity (28%)/ ATP binding (28%)	viral reproduction (69%)	N.A.*	2	no	cytoplasmic membrane	AEG73767.1
	hypothetical protein	translation release factor activity (24%)	translational termination (24%)	membrane (42%)	1	yes	unknown	AEG72694.1
	hypothetical protein	signal transducer activity (59%)/ receptor activity (59%)	signal transduction (60%)	membrane (90%)	2	no	cytoplasmic membrane	AEG72554.1
	hypothetical protein	metalloendopeptidase activity (34%)/ metal ion binding (34%)	proteolysis (34%)/ pathogenesis (17%)	extracellular region (34%)	0	no	unknown	AEG73596.1
	hypothetical protein	metal ion binding (28%)	transport (18%)	membrane (50%)	2	no	cytoplasmic membrane	AEG72923.1
	hypothetical protein	metal ion binding (43%)/ DNA binding (35%)	transcription (36%)	cytoplasm (30%)	0	no	cytoplasmic	AEG73063.1

	hypothetical protein	DNA binding (27%)	transcription (27%)	membrane (51%)	0	no	unknown	AEG73391.1
	hypothetical protein	structural molecule activity (60%)	transport (17%)/ cell adhesion (15%)	cytoplasm (63%)/ cytoskeleton (63%)	1	no	cytoplasmic membrane	AEG72824.1
	hypothetical protein	nucleotide (49%)/ATP binding (31%)	translation (17%)	cytoplasm (36%)	1	no	cytoplasmic	AEG73591.1
	hypothetical protein	protein binding (36%)/ receptor activity (42%)	cell adhesion (31%)	membrane (50%)	1	no	cytoplasmic	AEG73116.1
	hypothetical protein	metallopeptidase activity (20%)	proteolysis (20%)	membrane (54%)	1	no	cytoplasmic	AEG73115.1
	hypothetical protein	protein binding (32%)	phosphate transport (19%)/ cell adhesion (19%)	cytoplasm (35%)	1	no	cytoplasmic	AEG73114.1
	hypothetical protein	RNA binding (99%)	evasion by virus of host immune response (99%)	cytoplasm (99%)/ chromosome (99%)	3	no	cytoplasmic membrane	AEG73017.1
	hypothetical protein	catalytic activity (56%)/ transferase (44%)	metabolic process (48%)	membrane (100%)	3	no	cytoplasmic membrane	AEG72385.1
	hypothetical protein	transaminase/isomerase (100%)	porphyrin byosynthetic process (100%)	cytoplasm (100%)	0	no	cytoplasmic	AEG72616.1
	hypothetical protein	nucleotide binding (34%)/ translation initiation factor activity (32%)	translational initiation (32%)/ pathogenesis (20%)	membrane (42%)	1	no	unknown	AEG73324.1
	hypothetical protein	N.A.*	N.A.*	N.A.*	2	no	cytoplasmic membrane	AEG73429.1
CDSs with predicted function (present in several bacteria species, but absent in *M. haemocanis* str. illinois)	type II site-specific deoxyribonuclease	Type II site-specific deoxyribonuclease activity (83%)	DNA restriction-modification system (83%)	membrane (11%)	0	no	cytoplasmic	AEG73510.1
	Cytosine-specific methyltransferase	DNA (cytosine-5-)-methyltransferase activity (99%)	DNA methylation (99%)/ DNA restriction-modification system (99%)	chromosome (7%)	0	no	unknown	AEG73555.1
	C-5 cytosine-specific DNA methylase	DNA (cytosine-5-)-methyltransferase activity (100%)	DNA methylation (100%)/ DNA restriction-modification system (100%)	chromosome (9%)	0	no	cytoplasmic	AEG73509.1

[a] ESG software (sequence similarity-based function prediction) [36].

[b] prediction using PSORTb version 3 software [31].

*** N.A.: no function assigned by ESG software.**
