# Supplementary material for: Mycoplasma haemocanis – the canine hemoplasma and its feline counterpart in the genomic era
Source: Vet Res. 2012 Sep 28;43(1):66. doi: 10.1186/1297-9716-43-66 (PMC3496576; doi:10.1186/1297-9716-43-66)
Supplement: Additional file 3 — Table S3. Tandem repeats* identified in the genome of Mycoplasma haemocanis strain Illinois. Complete list of variable tandem repeats, identified using Tandem repeats finder program, indicating their sequences and position in the genome of M. haemocanis. [file 1297-9716-43-66-S3.pdf]

Supplementary Table 3. Tandem repeats* identified in the genome of *Mycoplasma haemocanis* strain Illinois.

Repeat	Start Position in Mhc Genome	End Position in Mhc Genome	Pattern Size	Copy Number	Match Percentage	Array Length	Profile Length	Pattern	Array Sequence
1	68251	68275	9	2.777778	100	25	9	AAAGCATCG	AAAGCATCGAAAGCATCGAAAGCAT
2	113109	113139	3	10.333333	100	31	3	TTC	TTCTTCTTCTTCTTCTTCTTCTTCTTCTTCT
3	113496	113544	6	8.166667	97	49	6	AGACCA	AGACCAAGACCAAGACCAAGACCAAGACCAAGACCAAGACCAAGGCCAA
4	192936	192972	6	6.166667	100	37	6	AAACCC	AAACCCAAACCCAAACCCAAACCCAAACCCAAACCCA
5	193201	193225	6	4.166667	100	25	6	ATCTTC	ATCTTCATCTTCATCTTCATCTTCA
6	193323	193354	6	5.333333	96	32	6	CATCAA	CATCAACATCAACATCAACATCAACAACAACA
7	352527	352567	11	3.727273	100	41	11	TAAAGGCGTTT	TAAAGGCGTTTTAAAGGCGTTTTAAAGGCGTTTTAAAGGCG
8	363904	363942	10	3.9	100	39	10	TCCAAACTAC	TCCAAACTACTCCAAACTACTCCAAACTACTCCAAACTA
9	382025	382055	11	2.818182	100	31	11	TAAAGACAAAC	TAAAGACAAACTAAAGACAAACTAAAGACAA
10	397671	397698	7	4	100	28	7	CAACTCT	CAACTCTCAACTCTCAACTCTCAACTCT
11	412296	412346	6	8.5	90	51	6	AGAAGA	AGAAGAGGAAGAAGAGGAAGAAGAGGAAGAAGAGGAAGAAGAGGAAGAAGA
12	412296	412349	9	6	100	54	9	AGAAGAGGA	AGAAGAGGAAGAAGAGGAAGAAGAGGAAGAAGAGGAAGAAGAGGAAGAAGAGGA
13	433693	433731	10	3.9	100	39	10	ACAGATAATT	ACAGATAATTACAGATAATTACAGATAATTACAGATAAT
14	436520	436551	12	2.666667	100	32	12	AGGGAGATCGAA	AGGGAGATCGAAAGGGAGATCGAAAGGGAGAT
15	438731	438766	12	2.923077	97	36	13	AGGAGCTAAGAG	AGGAGCTAAGAGAGGAGCTAAGAGAGGAGCTGAAGA
16	442467	442523	12	4.75	100	57	12	CAAGAGTTAAGA	CAAGAGTTAAGACAAGAGTTAAGACAAGAGTTAAGACAAGAGTTAAGACAAGAGTTA
17	445882	445906	8	3.125	100	25	8	TAGCTACC	TAGCTACCTAGCTACCTAGCTACCT
18	478337	478361	9	2.777778	100	25	9	ATAGCAAAG	ATAGCAAAGATAGCAAAGATAGCAA
19	501126	501150	9	2.777778	100	25	9	CGAAAAAGA	CGAAAAAGACGAAAAAGACGAAAAA
20	503698	503723	9	2.888889	100	26	9	AAGAAGTAG	AAGAAGTAGAAGAAGTAGAAGAAGTA
21	630263	630295	12	2.75	100	33	12	TAAGGGGTCTGA	TAAGGGGTCTGATAAGGGGTCTGATAAGGGGTC
22	635502	635542	16	2.705882	90	41	17	TAATCCTCCCAGCACC	TAATCCTCCCAGACCAACTCCTCCAGCACCTAATCCTCCCA
23	651566	651591	9	2.888889	100	26	9	AAAGGGGTG	AAAGGGGTGAAAGGGGTGAAAGGGGT
24	671591	671616	6	4.333333	100	26	6	AAGAGG	AAGAGGAAGAGGAAGAGGAAGAGGAA
25	673774	673825	9	5.777778	100	52	9	ATTGGATGA	ATTGGATGAATTGGATGAATTGGATGAATTGGATGAATTGGATGAATTGGAT
26	674500	674524	6	4.166667	100	25	6	GGAAGA	GGAAGAGGAAGAGGAAGAGGAAGAG
27	674752	674776	9	2.777778	100	25	9	GGTTTGTGA	GGTTTGTGAGGTTTGTGAGGTTTGT
28	680483	680507	6	4.166667	100	25	6	TTGTGC	TTGTGCTTGTGCTTGTGCTTGTGCT
29	692026	692051	6	4.333333	100	26	6	TGGTTG	TGGTTGTGGTTGTGGTTGTGGTTGTG
30	694302	694359	6	9.666667	98	58	6	TGTACC	TGTACCTGTACCTGTACCTGTACCTGTACCTGTACCTGTACCTGTACCTGTAACTGTA
31	736969	736994	9	2.888889	100	26	9	ACGAAAAAG	ACGAAAAAGACGAAAAAGACGAAAAA
32	846032	846137	12	8.833333	100	106	12	ATCTTCGGCTTT	ATCTTCGGCTTTATCTTCGGCTTTATCTTCGGCTTTATCTTCGGCTTTATCTTCGGCTTTATCTTCGGCTTTATCTTCGGCTTTATCTTCGGCTTTATCTTCGGCT
33	886587	886612	6	4.333333	100	26	6	TGCTGG	TGCTGGTGCTGGTGCTGGTGCTGGTG

***TRDB - The Tandem Repeats Database, Nucleic Acids Research, 2006, Vol. 00, Database issue D1-D8 doi:10.1093/nar/gkl1013. Parameters: match, 2, mismatch, 7, indels, 2.**

Mhc: *Mycoplasma haemocanis* strain Illinois
